# Adrenal involvement in antiphospholipid syndrome and SLE-spectrum disease: a systematic review and pooled individual-patient analysis

**DOI:** 10.3389/fimmu.2026.1851029

**Published:** 2026-06-03

**Authors:** Sheng-Guang Li, Ting Long, Ruohan Yu, Lina Zhang, Jing Zhang, Ji Li, Yadan Zou, Yanfeng Zhang

**Affiliations:** Department of Rheumatology and Immunology, Peking University International Hospital, Beijing, China

**Keywords:** adrenal hemorrhage, adrenal insufficiency, antiphospholipid syndrome, catastrophic antiphospholipid syndrome, pooled analysis, systematic review, systemic lupus erythematosus, thrombosis

## Abstract

**Background:**

Adrenal involvement is a rare but potentially severe immunothrombotic manifestation of antiphospholipid syndrome (APS) and systemic lupus erythematosus (SLE)-spectrum disease. Because the term adrenal crisis should be reserved for patients with acute adrenal insufficiency accompanied by hypotension or shock, this review distinguishes adrenal vascular injury, adrenal insufficiency, and strict adrenal crisis.

**Methods:**

We performed a systematic review and individual-patient pooled analysis of published cases of APS/SLE-spectrum adrenal involvement through March 31, 2026. The initial database search was performed from inception to February 10, 2026 and was updated through March 31, 2026. The study selection followed PRISMA 2020 principles, and the protocol was registered in PROSPERO (CRD420261298329). Published patients were harmonized for diagnosis stratum, precipitating trigger, adrenal imaging phenotype, adrenal insufficiency chronology, immunologic profile, thrombotic burden, treatment/follow-up variables, CAPS presentation context, and outcomes.

**Results:**

A total of 103 studies comprising 155 published patients were included; 143 entered the main analysis set. The median age was 43.8 years, 49.0% were female, and primary APS accounted for 67.1% of the main cohort. Hemorrhage-dominant adrenal injury was the leading imaging phenotype (66.9%), bilateral adrenal involvement was highly prevalent (86.3% of informative cases), and adrenal insufficiency was the first manifestation in 73.6% of informative cases. Among codable main-analysis cases, hypotension was documented in 60/125 (48.0%), strict adrenal crisis in 64/124 (51.6%), hyponatremia in 47/142 (33.1%), and hyperkalemia in 57/134 (42.5%). CAPS was treated as a presentation/severity context rather than as a downstream consequence and was reported in 59.8% of evaluable patients.

**Conclusion:**

Adrenal involvement in APS and lupus-spectrum disease is a heterogeneous but clinically recognizable high-risk phenotype characterized by bilateral adrenal injury, hemorrhagic predominance, frequent sentinel presentation as adrenal insufficiency, and substantial CAPS burden. Careful terminology helps separate adrenal vascular injury, adrenal insufficiency, and strict adrenal crisis while preserving the need for urgent endocrine rescue and systemic thrombotic evaluation.

**Systematic review registration:**

https://www.crd.york.ac.uk/prospero/, identifier CRD420261298329.

## Introduction

Antiphospholipid syndrome (APS) is a systemic autoantibody-mediated thrombo-inflammatory disorder defined by arterial and/or venous thrombosis or pregnancy morbidity in the setting of persistent antiphospholipid antibodies (aPL) (1, 2). APS may occur as a primary disease or in association with systemic lupus erythematosus (SLE), the prototypic multisystem autoimmune disease in which hematologic abnormalities, immune thrombocytopenia, and thrombotic complications contribute substantially to morbidity (2–4). Across the APS–SLE interface, clinically relevant phenotypes are shaped not only by aPL profile but also by the broader inflammatory, endothelial, and microvascular context in which thrombosis develops.

Adrenal involvement is an uncommon but clinically consequential manifestation within this spectrum. In contrast to autoimmune adrenalitis, tuberculosis, and other classic causes of primary adrenal insufficiency, APS/SLE-related adrenal failure typically reflects hemorrhagic infarction, venous thrombosis, microthrombosis, or necrotic vascular injury of the adrenal glands (5–10). This framing is consistent with the 2023 ACR/EULAR APS classification criteria, in which adrenal hemorrhage or microthrombosis is included in the microvascular clinical domain (10). The adrenal circulation is uniquely vulnerable because a rich arterial plexus drains through limited venous outflow, creating a setting in which thrombotic obstruction can propagate into hemorrhage and cortical destruction (9).

Important APS-focused adrenal series have already established that this phenotype is rare but severe. Espinosa et al. summarized 86 patients with adrenal involvement in APS (6), and Meade-Aguilar et al. recently reported a mixed multicenter cohort and systematic literature review of 256 patients with APS-associated adrenal hemorrhage (11). Those studies showed frequent bilateral involvement, adrenal insufficiency, abdominal symptoms, electrolyte abnormalities, and mortality. Some other reports, including cases arising in SLE-associated immune thrombocytopenia and treatment-related prothrombotic contexts, suggest that trigger context, adrenal imaging pattern, and systemic thrombotic burden may meaningfully influence severity and outcome (12–16). The present study was therefore reframed not as the first large synthesis of adrenal hemorrhage but as a complementary individual-patient analysis that extends prior APS-focused work by harmonizing APS/SLE-spectrum diagnostic strata, trigger context, adrenal imaging patterns, CAPS presentation context, and case-level follow-up variables.

In this study, we performed a systematic review and individual-patient pooled analysis of published cases of adrenal involvement in APS and SLE-spectrum disease. Our aims were to define the clinical and immunologic phenotype, characterize precipitating triggers and adrenal imaging patterns, examine the frequency with which adrenal insufficiency was the sentinel presentation, summarize CAPS as a presentation/severity context, describe long-term adrenal replacement and mortality where reported, and derive a pragmatic clinical recognition framework.

## Methods

### Study design and reporting

We performed a systematic review of published cases of adrenal involvement occurring in the setting of APS, SLE, or related SLE-spectrum connective tissue disease. The study was designed as an individual-patient pooled analysis of published case reports and case series. Reporting and study selection were structured according to PRISMA 2020 principles (17). The review protocol was prospectively registered in the PROSPERO database (CRD420261298329).

### Literature search and study selection

A structured literature search was conducted in PubMed, Embase, and Web of Science from database inception to February 10, 2026 and was updated through March 31, 2026. Full reproducible search strategies for each database are provided in **Supplementary Appendix 1**. Search concepts captured APS/aPL, SLE or lupus-spectrum connective tissue disease, adrenal hemorrhage, adrenal infarction, adrenal thrombosis or microthrombosis, adrenal necrosis, adrenal enlargement compatible with vascular injury, and adrenal insufficiency. Addison disease was used only as a historical free-text synonym because older reports frequently labeled vascular primary adrenal insufficiency as Addison disease; classic autoimmune Addison disease without APS/SLE-spectrum disease and vascular adrenal involvement was excluded during screening.

### Eligibility framework and analytic datasets

All eligible published patients were first entered into the full review dataset. A main analysis set was then defined *a priori* by excluding non-target phenotype cases and sensitivity-only cases. Because many source reports predated current classification frameworks, case adjudication was based on the published clinical and laboratory record, anchored pragmatically to the revised Sapporo APS criteria and interpreted considering the 2023 ACR/EULAR APS classification concepts, including adrenal hemorrhage or microthrombosis as a microvascular APS manifestation when appropriate (1, 10). Definite APS required source-level clinical APS features with reported aPL positivity compatible with APS classification, probable APS denoted cases with strong clinical APS phenotype but incomplete laboratory or persistence documentation, and non-APS SLE-spectrum disease denoted SLE/CTD cases with adrenal vascular involvement but no source-level APS classification.

### Data extraction and variable harmonization

Patient-level variables were extracted independently by two reviewers (Sheng-Guang Li and Ting Long) and harmonized into a prespecified case report form; disagreements were resolved by discussion with senior review of the source report. For title/abstract screening, reviewer A selected 109/1, 024 records for full-text assessment and reviewer B selected 108/1, 024; 15 records were discordant before consensus, yielding an observed agreement of 98.5% and Cohen’s kappa of 0.923. For full-text eligibility assessment, reviewer A included 103/107 reports and reviewer B included 102/107; one report was discordant before consensus, yielding an observed agreement of 99.1% and Cohen’s kappa of 0.884. Extracted domains included demographic characteristics, diagnosis stratum, precipitating factor, electrolyte abnormalities, adrenal chronology, symptoms, hemodynamic status, immunologic profile, hematologic findings, adrenal imaging phenotype, thrombotic burden, treatment, follow-up imaging, and clinical outcomes. Imaging variables were extracted from the source authors’ radiology descriptions; no adrenal radiologist independently re-read the original imaging.

### Outcomes

CAPS was analyzed as a clinical presentation/severity context rather than as a downstream outcome of adrenal involvement. Outcomes were death, long-term or permanent adrenal replacement, and adrenal recovery; severe composite summaries were retained only as exploratory descriptive signals. Because outcome reporting was incomplete in many reports, all proportions were calculated using available-case denominators rather than the full cohort denominator.

### Statistical analysis

Continuous variables were summarized as median with interquartile range (IQR) and categorical variables as number and percentage. Percentages were based on available cases for each variable. Comparisons across diagnosis strata were considered exploratory because of sparse cells and marked imbalance in subgroup size; Kruskal–Wallis test was used for continuous variables and chi-square-based comparisons for categorical variables, with Fisher’s exact test used when appropriate. Multivariable modeling was performed using Firth bias-reduced logistic regression to address small-sample and rare-event bias (18–20).

Hyponatremia and hyperkalemia were coded from source-reported abnormalities or, when numeric values were available, as sodium <135 mmol/L and potassium >5.0 mmol/L, respectively. Adrenal insufficiency required source-reported primary adrenal insufficiency/Addison disease or low cortisol with elevated ACTH/clinical steroid replacement. Strict adrenal crisis required adrenal insufficiency accompanied by hypotension, shock, circulatory collapse, or an explicit source diagnosis of adrenal crisis. aPL positivity and triple positivity were coded according to source-reported laboratory positivity; criterion-level or persistent positivity was retained when explicitly documented.

## Results

### Study selection

**Figure 1:**
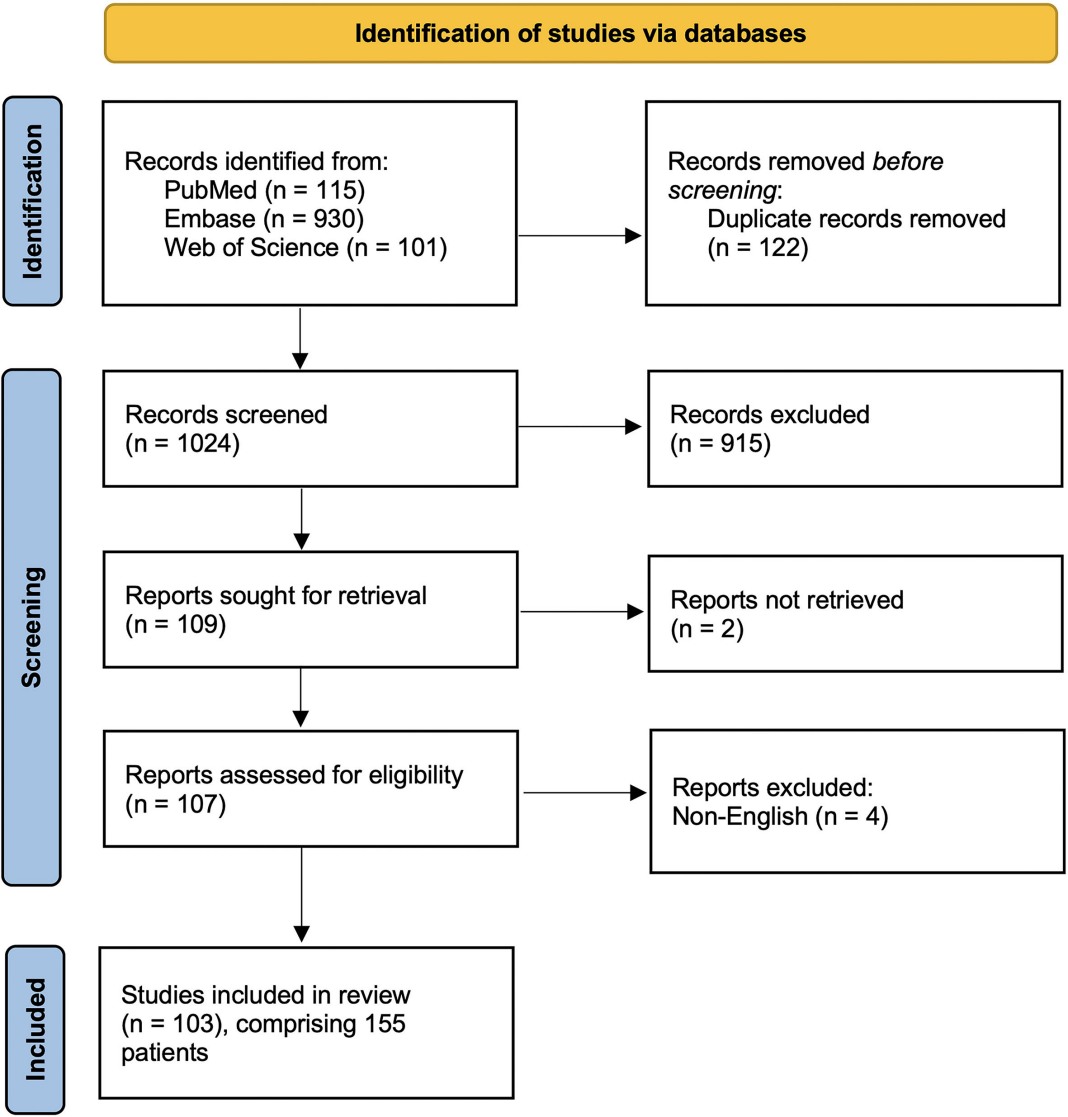
PRISMA 2020 flow diagram of study selection. PRISMA 2020 flow diagram showing the identification, screening, retrieval, eligibility assessment, and final inclusion of studies. A total of 1, 146 records were identified through database searching. After the removal of 122 duplicates, 1, 024 records underwent title and abstract screening, of which 915 were excluded. Among the 109 reports sought for retrieval, two could not be retrieved and four non-English reports were excluded. Ultimately, 103 studies comprising 155 published patients were included.

A total of 1, 146 records were identified through database searching, including 115 from PubMed, 930 from Embase, and 101 from Web of Science. After the removal of 122 duplicate records, 1, 024 records underwent title and abstract screening, of which 915 were excluded. Among the 109 reports sought for retrieval, two could not be retrieved and four non-English reports were excluded. Ultimately, 103 studies were included in the final review, yielding 155 published patients (**Figure 1**). Of the included studies, 56 were published before 2000 (21–76) and 47 were published in or after 2000 (6, 8, 77–113).

### Composition of the analytical dataset

**Figure 2:**
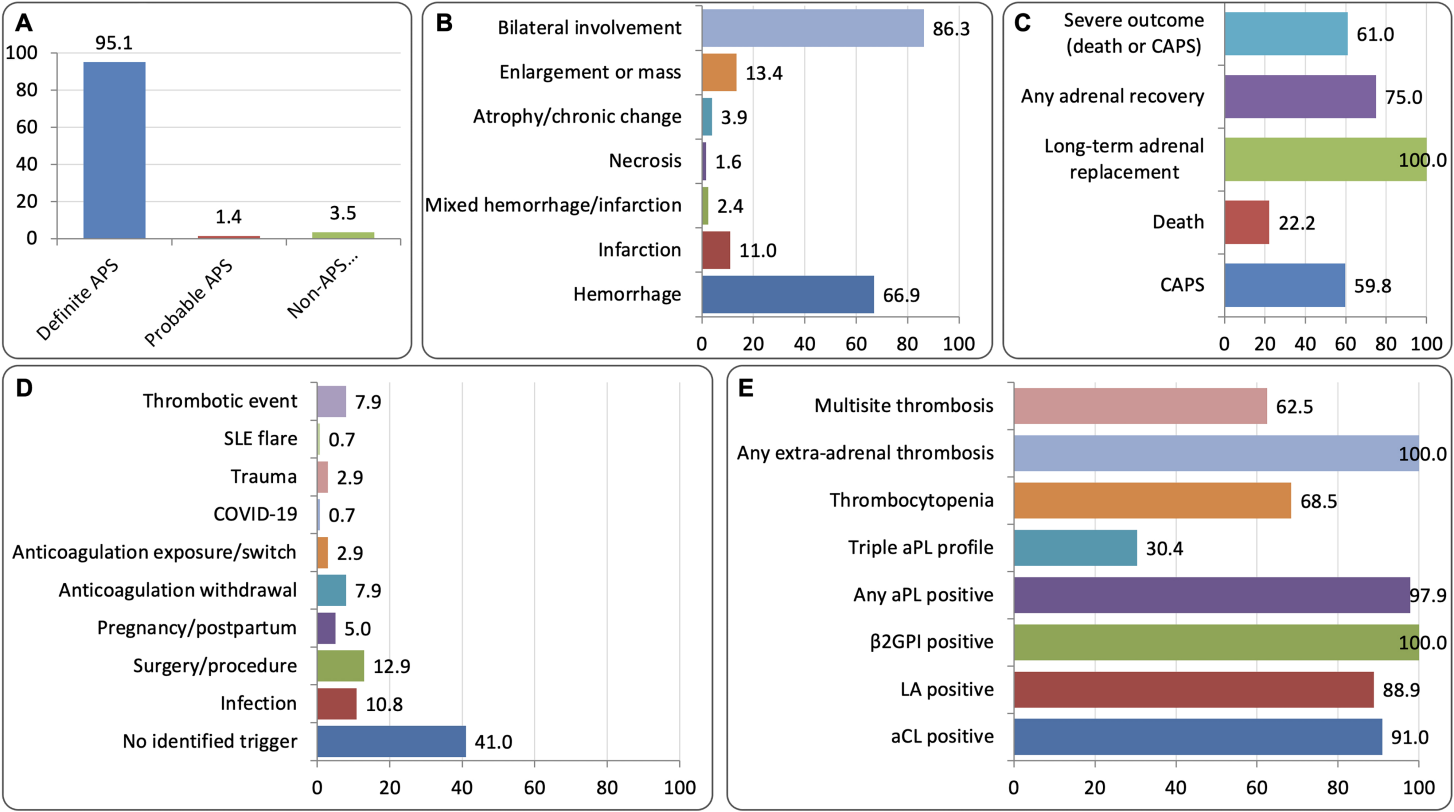
Phenotypic composition of the main analysis set. Phenotypic overview of the main analysis set. **(A)** APS research classification. **(B)** Adrenal imaging phenotypes. **(C)** Clinical severity and follow-up outcomes, including CAPS, death, long-term adrenal replacement, adrenal recovery, and severe composite outcome. **(D)** Precipitating factors. **(E)** Immuno-thrombotic burden. **(A)** uses the full main-analysis denominator, whereas **(B–E)** use available-case denominators for each variable.

After exclusion of non-target phenotype cases and sensitivity-only cases, the main analysis set comprised 143 patients; the remaining 12 patients were retained outside the primary analytic dataset because of incomplete diagnostic certainty, atypical adrenal phenotype, or insufficient source detail for primary classification. The phenotypic composition of the main analysis set is shown in **Figure 2**. Most patients belonged to the definite APS category, whereas probable APS and non-APS SLE-spectrum disease accounted for only a small minority.

### Baseline characteristics of the main analysis set

Baseline characteristics are summarized in **Table 1**. The median age of the main analysis set was 43.8 years (IQR 32.0–54.0), and 70/143 patients (49.0%) were female. Primary APS was the dominant diagnostic subgroup (96/143, 67.1%), followed by secondary APS associated with SLE (36/143, 25.2%). Isolated SLE and other CTD-associated APS were uncommon. Age ≥50 years was present in 50/143 patients (35.0%).

**Table 1:** Baseline clinical, immunologic, imaging, and outcome characteristics of the main analysis set.

Characteristic	Overall	Definite APS	Probable APS	Non-APS SLE-spectrum
Cases, *n*	143	136	2	5
Female sex	70 (49.0%)	66 (48.5%)	1 (50.0%)	3 (60.0%)
Age, years	43.8 (32.0–54.0)	44.0 (32.1–54.2)	30.4 (30.2–30.7)	35.0 (32.0–49.0)
Age, ≥50 years	50 (35.0%)	49 (36.0%)	0 (0.0%)	1 (20.0%)
Diagnosis stratum				
Primary APS	96 (67.1%)	96 (70.6%)	0 (0.0%)	0 (0.0%)
Secondary APS (SLE-associated)	36 (25.2%)	36 (26.5%)	0 (0.0%)	0 (0.0%)
Isolated SLE	5 (3.5%)	0 (0.0%)	0 (0.0%)	5 (100.0%)
Other CTD-associated APS	4 (2.8%)	4 (2.9%)	0 (0.0%)	0 (0.0%)
Precipitating factor				
Trigger information available	139 (100.0%)	132 (100.0%)	2 (100.0%)	5 (100.0%)
Infection	15 (10.8%)	15 (11.4%)	0 (0.0%)	0 (0.0%)
Surgery/procedure	18 (12.9%)	15 (11.4%)	2 (100.0%)	1 (20.0%)
Pregnancy/postpartum	7 (5.0%)	5 (3.8%)	1 (50.0%)	1 (20.0%)
Anticoagulation withdrawal	11 (7.9%)	11 (8.3%)	0 (0.0%)	0 (0.0%)
Anticoagulation exposure/switch	4 (2.9%)	4 (3.0%)	0 (0.0%)	0 (0.0%)
COVID-19	1 (0.7%)	1 (0.8%)	0 (0.0%)	0 (0.0%)
Trauma	4 (2.9%)	4 (3.0%)	0 (0.0%)	0 (0.0%)
SLE flare	1 (0.7%)	0 (0.0%)	0 (0.0%)	1 (20.0%)
Clinical presentation				
Hyponatremia	47 (33.1%)	45 (33.3%)	0 (0.0%)	2 (40.0%)
Hyperkalemia	57 (42.5%)	56 (43.4%)	0 (0.0%)	1 (33.3%)
CAPS	49 (59.8%)	49 (63.6%)	0 (0.0%)	0 (0.0%)
Addison/adrenal insufficiency as first manifestation	67 (73.6%)	65 (76.5%)	2 (100.0%)	0 (0.0%)
Classic electrolyte pattern	21 (15.7%)	21 (16.3%)	0 (0.0%)	0 (0.0%)
Immunologic profile				
aCL-positive	121 (91.0%)	119 (93.7%)	2 (100.0%)	0 (0.0%)
LA-positive	120 (88.9%)	116 (90.6%)	2 (100.0%)	2 (40.0%)
ANA-positive	65 (52.0%)	61 (51.3%)	0 (0.0%)	4 (100.0%)
Anti-dsDNA-positive	27 (23.7%)	25 (22.9%)	0 (0.0%)	2 (66.7%)
β2GPI-positive	6 (100.0%)	6 (100.0%)	NR	NR
Any aPL-positive	140 (97.9%)	133 (97.8%)	2 (100.0%)	5 (100.0%)
Triple aPL-profile	38 (30.4%)	38 (31.9%)	0 (0.0%)	0 (0.0%)
Hematologic profile				
Thrombocytopenia	61 (68.5%)	58 (69.0%)	1 (100.0%)	2 (50.0%)
Imaging phenotype				
Bilateral adrenal involvement	82 (86.3%)	80 (87.0%)	NR	2 (66.7%)
Hemorrhage	85 (66.9%)	82 (68.3%)	0 (0.0%)	3 (60.0%)
Infarction	14 (11.0%)	14 (11.7%)	0 (0.0%)	0 (0.0%)
Mixed hemorrhage/infarction	3 (2.4%)	3 (2.5%)	0 (0.0%)	0 (0.0%)
Necrosis	2 (1.6%)	2 (1.7%)	0 (0.0%)	0 (0.0%)
Atrophy/chronic change	5 (3.9%)	5 (4.2%)	0 (0.0%)	0 (0.0%)
Enlargement or mass	17 (13.4%)	16 (13.3%)	1 (50.0%)	0 (0.0%)
Thrombotic burden and outcomes				
Any extra-adrenal thrombosis	8 (100.0%)	7 (100.0%)	NR	1 (100.0%)
Multisite thrombosis	5 (62.5%)	4 (57.1%)	NR	1 (100.0%)
Long-term adrenal replacement	3 (100.0%)	3 (100.0%)	NR	NR
Any adrenal recovery	3 (75.0%)	3 (75.0%)	NR	NR
Death	2 (22.2%)	1 (12.5%)	NR	1 (100.0%)
Severe outcome (death or CAPS)	50 (61.0%)	49 (63.6%)	0 (0.0%)	1 (25.0%)

Data are presented as median (IQR) for continuous variables and *n* (%) for categorical variables. Percentages were calculated from available cases for each variable. Thrombocytopenia was summarized as the proportion of reported low platelet counts because the exact numeric platelet values were unavailable in most published cases. The main analysis set excluded non-target phenotype cases and sensitivity-only cases. CAPS, catastrophic antiphospholipid syndrome; aCL, anticardiolipin antibodies; LA, lupus anticoagulant; β2GPI, anti-β2 glycoprotein I.

### Trigger profile and clinical presentation

When precipitating factor categories were harmonized across reports, a specific upstream context was identified in many cases, although “none identified” remained common. Among the main analysis set, infection was reported in 15/139 informative cases (10.8%), surgery or invasive procedures in 18/139 (12.9%), pregnancy/postpartum in 7/139 (5.0%), anticoagulation withdrawal in 11/139 (7.9%), anticoagulation exposure or switching in 4/139 (2.9%), trauma in 4/139 (2.9%), and overt SLE flare in 1/139 (0.7%). Electrolyte disturbances were frequent among evaluable cases, with hyponatremia reported in 47/142 (33.1%) and hyperkalemia in 57/134 (42.5%). Addison disease or adrenal insufficiency as the first manifestation of APS/SLE-spectrum disease was documented in 67/91 informative cases (73.6%).

To directly address the adrenal-involvement characteristics requested during review, we performed structured extraction of symptom, hemodynamic, endocrine, treatment, and follow-up imaging variables from the case-level table and source-verification fields (**Table 2**). Among the 143 main-analysis cases, codable symptom fields showed abdominal pain in 52/125 (41.6%), fever in 44/125 (35.2%), weakness/fatigue/malaise/lethargy in 44/125 (35.2%), nausea/vomiting in 34/125 (27.2%), flank/back pain in 14/125 (11.2%), and hyperpigmentation in 12/125 (9.6%). Hypotension was documented in 60/125 (48.0%), shock or circulatory collapse in 3/125 (2.4%), and strict adrenal crisis in 64/124 (51.6%). Cortisol and ACTH values were reported in 102/143 (71.3%) and 67/143 (46.9%) main-analysis cases, respectively; raw values were retained in the extraction file but not meta-analyzed because timing, assay units, and stimulation status varied across reports.

**Table 2:** Structured clinical, endocrine, treatment, imaging, and CAPS-context variables requested during review.

Variable	Main-analysis summary	Denominator/comment
Abdominal pain	52/125 (41.6%)	Codable symptom fields; 18 NR/unclear
Fever	44/125 (35.2%)	Codable symptom fields; 18 NR/unclear
Weakness/fatigue/malaise/lethargy	44/125 (35.2%)	Codable symptom fields; 18 NR/unclear
Nausea/vomiting	34/125 (27.2%)	Codable symptom fields; 18 NR/unclear
Flank/back pain	14/125 (11.2%)	Codable symptom fields; 18 NR/unclear
Hyperpigmentation	12/125 (9.6%)	Codable symptom fields; 18 NR/unclear
Altered mental status/confusion	10/125 (8.0%)	Codable symptom fields; 18 NR/unclear
Hypotension	60/125 (48.0%)	Source-reported hypotension, low BP, or orthostatic/postural hypotension
Shock/circulatory collapse	3/125 (2.4%)	Source-reported shock, collapse, or hemodynamic instability
Strict adrenal crisis	64/124 (51.6%)	Adrenal insufficiency plus hypotension/shock/circulatory instability or explicit adrenal crisis
Hyponatremia	47/142 (33.1%)	Source-reported or sodium <135 mmol/L when numeric value was available
Hyperkalemia	57/134 (42.5%)	Source-reported or potassium >5.0 mmol/L when numeric value was available
Cortisol value reported	102/143 (71.3%)	Raw values retained; timing, units, and stimulation status varied
ACTH value reported	67/143 (46.9%)	Raw values retained; timing and units varied
Bilateral adrenal involvement on imaging	82/95 (86.3%)	Based on source-report CT/MRI descriptions
Follow-up adrenal imaging specifically described	3/143 (2.1%)	Very sparse reporting; interpreted descriptively only
Long-term adrenal replacement follow-up codable	3/143 (2.1%)	Not a prevalence estimate; many reports used nonspecific steroid/adrenal replacement wording
Glucocorticoid vs. mineralocorticoid regimen	Not reliably pooled	Regimen separated only when hydrocortisone/fludrocortisone or equivalent was explicitly documented
CAPS as presentation/context	49/82 (59.8%)	Presentation/severity context, not a downstream outcome

Data are shown as *n*/*N* (%) unless otherwise indicated. Percentages were calculated from available cases. Treatment and follow-up imaging variables were interpreted descriptively because source reports often used nonspecific treatment wording and rarely reported structured follow-up imaging.

### Immunologic and hematologic profile

Antiphospholipid antibody positivity was a dominant feature of the cohort. aCL positivity was reported in 121/133 informative patients (91.0%) and LA positivity in 120/135 (88.9%). Nearly all informative cases had at least one positive antiphospholipid antibody (140/143, 97.9%). ANA positivity was common (65/125, 52.0%), whereas anti-dsDNA positivity was less frequent (27/114, 23.7%). β2GPI positivity was documented in only six informative patients (**Table 1**), reflecting marked under-reporting of this variable in the case-report literature. Thrombocytopenia was also frequent; because exact platelet values were often unavailable, it was summarized descriptively as reported low platelet count and was present in 61/89 evaluable patients (68.5%).

### Imaging phenotype, adrenal chronology, and thrombotic burden

Adrenal hemorrhage was the dominant imaging phenotype in the main analysis set, occurring in 85/127 informative cases (66.9%). Infarction was less frequent (14/127, 11.0%), with mixed hemorrhage/infarction, necrosis, atrophy/chronic change, and enlargement/mass representing smaller subsets. Bilateral adrenal involvement was reported in 82/95 informative patients (86.3%), indicating that adrenal involvement in APS/SLE-spectrum disease most often affected both glands (**Table 1**). Where specifically described, extra-adrenal thrombosis was common and often multisite, supporting the interpretation that adrenal involvement frequently occurred within a broader systemic thrombotic phenotype rather than as an isolated adrenal event.

### Outcomes

Outcome reporting was incomplete across published cases. Long-term adrenal replacement, adrenal recovery, and follow-up adrenal imaging were sparsely but clinically important variables. Available reports suggested that durable hormone replacement was common once overt adrenal failure had occurred, whereas complete adrenal recovery was uncommon; however, these estimates were limited by denominator instability and inconsistent reporting of glucocorticoid versus mineralocorticoid replacement. Mortality was documented in a limited number of informative cases and was therefore interpreted separately and cautiously.

### Comparison by diagnosis stratum

Descriptive comparisons across diagnosis strata are provided in **Supplementary Table 1** rather than emphasized in the main text because the dataset was strongly weighted toward primary APS and secondary APS associated with SLE, while isolated SLE, other CTD-associated APS, and possible SLE-spectrum APS were sparsely represented. These subgroup comparisons remain descriptive because of sparse cells and uneven group sizes.

### Cases in which adrenal insufficiency was the first manifestation

**Table 3** summarizes the patient-level details of published cases in which adrenal insufficiency or Addison disease represented the sentinel manifestation of APS/SLE-spectrum disease. These cases spanned more than four decades and were dominated by primary APS and secondary APS associated with SLE. Hemorrhage-dominant imaging was the most frequent adrenal phenotype, although infarction—dominant, enlargement/mass, imaging—unclear, and normal/other phenotypes also occurred. Collectively, these cases illustrate an important sentinel presentation in which adrenal insufficiency preceded formal recognition of the systemic autoimmune or thrombotic disorder.

**Table 3:** Published cases in which Addison disease or adrenal insufficiency was the first manifestation of APS/SLE-spectrum disease.

Patient (Ref.)	Year	Diagnosis	Trigger	Imaging phenotype
Mueh JR	1980	PAPS	Surgery/procedure	Imaging unclear
Bendon RW	1987	Secondary APS (SLE-associated)	Pregnancy/postpartum	Imaging unclear
Grottolo A	1988	PAPS	Surgery/procedure	Normal/other
Pelkonen P	1988	PAPS	None identified	Imaging unclear
Alperin N	1989	PAPS	Thrombosis event	Hemorrhage-dominant
Asherson RA	1989	PAPS	Multiple triggers	Necrosis/chronic change
Carette S	1989	PAPS	Other/unspecified trigger	Imaging unclear
Rao RH	1989	PAPS	None identified	Hemorrhage-dominant
Yap AS, case 1	1989	PAPS	None identified	Hemorrhage-dominant
Yap AS, case 2	1989	PAPS	None identified	Enlargement/mass
Yap AS, case 3	1989	Other CTD-associated APS	None identified	Enlargement/mass
Carlisle EJF	1990	Secondary APS (SLE-associated)	Multiple triggers	Enlargement/mass
Levy EN, case 1	1990	Possible SLE-spectrum APS	Multiple triggers	Normal/other
Levy EN, case 2	1990	Secondary APS (SLE-associated)	None identified	Hemorrhage-dominant
Rose C	1990	PAPS	None identified	Imaging unclear
Siu SCB, case 1	1990	Secondary APS (SLE-associated)	None identified	Enlargement/mass
Siu SCB, case 2	1990	Secondary APS (SLE-associated)	Surgery/procedure	Hemorrhage-dominant
Siu SCB, case 3	1990	PAPS	None identified	Hemorrhage-dominant
Walz B	1990	PAPS	None identified	Hemorrhage-dominant
Al-Momen AK, case 1	1991	PAPS	None identified	Normal/other
Al-Momen AK, case 2	1991	Secondary APS (SLE-associated)	None identified	Enlargement/mass
Dorling A	1991	PAPS	Infection	Imaging unclear
Inam S	1991	Secondary APS (SLE-associated)	None identified	Infarction-dominant
Komesaroff PA	1991	PAPS	Surgery/procedure	Infarction-dominant
Lenaerts J	1991	Secondary APS (SLE-associated)	None identified	Hemorrhage-dominant
McCroskey RD, case 1	1991	PAPS	None identified	Hemorrhage-dominant
McCroskey RD, case 2	1991	PAPS	None identified	Hemorrhage-dominant
McCroskey RD, case 3	1991	Isolated SLE	None identified	Hemorrhage-dominant
McCroskey RD, case 4	1991	Secondary APS (SLE-associated)	None identified	Hemorrhage-dominant
Ames DE	1992	PAPS	Multiple triggers	Infarction-dominant
Da Costa GM	1992	Secondary APS (SLE-associated)	Infection	Hemorrhage-dominant
Dagenais P	1992	Secondary APS (SLE-associated)	None identified	Hemorrhage-dominant
Perez RE	1992	PAPS	None identified	Enlargement/mass
Ghosh S	1993	PAPS	None identified	Imaging unclear
Le Thi Huong Du, case 1	1993	PAPS	None identified	Hemorrhage-dominant
Le Thi Huong Du, case 2	1993	Secondary APS (SLE-associated)	None identified	Hemorrhage-dominant
Le Thi Huong Du, case 3	1993	Secondary APS (SLE-associated)	None identified	Hemorrhage-dominant
Le Thi Huong Du, case 4	1993	PAPS	Trauma	Hemorrhage-dominant
Roujanski C	1993	PAPS	Surgery/procedure	Hemorrhage-dominant
Vengrove MA	1993	PAPS	Multiple triggers	Hemorrhage-dominant
Brucato A	1994	PAPS	None identified	Imaging unclear
Cappell MS	1994	PAPS	None identified	Imaging unclear
Christofilis MA	1994	PAPS	Surgery/procedure	Hemorrhage-dominant
Hochfeld M	1994	PAPS	Pregnancy/postpartum	Imaging unclear
Perret C	1994	PAPS	None identified	Infarction-dominant
Rigalleau V	1994	Secondary APS (SLE-associated)	None identified	Hemorrhage-dominant
Ruffatti A	1994	PAPS	None identified	Hemorrhage-dominant
Arnason JA	1995	Secondary APS (SLE-associated)	Infection	Normal/other
Dubois A	1995	PAPS	Other/unspecified trigger	Necrosis/chronic change
Gaspari JC	1995	PAPS	Infection	Hemorrhage-dominant
Luppi M	1995	PAPS	None identified	Infarction-dominant
Molina M	1995	PAPS	Infection	Hemorrhage-dominant
Papadopoulos KI	1995	PAPS	Surgery/procedure	Hemorrhage-dominant
Provenzale, case 1	1995	PAPS	None identified	Hemorrhage-dominant
Provenzale, case 2	1995	PAPS	Other/unspecified trigger	Hemorrhage-dominant
Provenzale, case 3	1995	PAPS	Other/unspecified trigger	Hemorrhage-dominant
Provenzale, case 4	1995	PAPS	Other/unspecified trigger	Hemorrhage-dominant
Provenzale, case 5	1995	PAPS	Other/unspecified trigger	Hemorrhage-dominant
Teyssier G	1995	PAPS	Infection	Hemorrhage-dominant
Gonzalez G	1996	PAPS	None identified	Hemorrhage-dominant
Guibal F	1996	PAPS	Pregnancy/postpartum	Hemorrhage-dominant
Horustzky A	1996	PAPS	None identified	Hemorrhage-dominant
Lai Y-P	1996	PAPS	None identified	Hemorrhage-dominant
Hsu B	1997	PAPS	Surgery/procedure	Enlargement/mass
Marie I	1997	PAPS	None identified	Infarction-dominant
Argento A	1998	PAPS	None identified	Infarction-dominant
Asherson RA	1998	Secondary APS (SLE-associated)	None identified	Imaging unclear
Caron P	1998	PAPS	None identified	Hemorrhage-dominant
Cerveny KC	1998	PAPS	None identified	Mixed hemorrhage/infarction
Philippe Caron	1998	PAPS	Other/unspecified trigger	Hemorrhage-dominant
Tauchmanov L	1998	PAPS	None identified	Hemorrhage-dominant
Jose MD	1999	PAPS	Surgery/procedure	Infarction-dominant
Jullien V	1999	PAPS	None identified	Hemorrhage-dominant
Cisternas M	2000	PAPS	Anticoagulation-related	Imaging unclear
Queralt C	2000	Secondary APS (SLE-associated)	None identified	Imaging unclear
Satta MA	2000	PAPS	None identified	Normal/other
Zoli A	2000	Secondary APS (SLE-associated)	Anticoagulation-related	Imaging unclear
Asherson RA, case 1	2001	PAPS	None identified	Infarction-dominant
Asherson RA, case 2	2001	PAPS	None identified	Imaging unclear
Asherson RA, case 3	2001	PAPS	Trauma	Imaging unclear
Asherson RA, case 4	2001	Secondary APS (SLE-associated)	Multiple triggers	Hemorrhage-dominant
Vella A	2001	PAPS	Infection	Hemorrhage-dominant
Vlot AJ	2001	PAPS	None identified	Infarction-dominant
Barreiro-Garcia G	2002	Secondary APS (SLE-associated)	Trauma	Hemorrhage-dominant
Yanni LM	2002	PAPS	Thrombosis event	Hemorrhage-dominant
Espinosa, case 1	2003	PAPS	Infection	Enlargement/mass
Espinosa, case 2	2003	Secondary APS (SLE-associated)	Infection	Enlargement/mass
Espinosa, case 3	2003	Secondary APS (SLE-associated)	Anticoagulation-related	Hemorrhage-dominant
Espinosa, case 4	2003	Secondary APS (SLE-associated)	Thrombosis event	Hemorrhage-dominant
Espinosa, case 5	2003	PAPS	None identified	Hemorrhage-dominant
Espinosa, case 6	2003	PAPS	None identified	Hemorrhage-dominant
Riddell	2004	Secondary APS (SLE-associated)	Other/unspecified trigger	Mixed hemorrhage/infarction
Bhattacharya	2005	PAPS	Other/unspecified trigger	Hemorrhage-dominant
Zhang ZL, case 1	2009	Isolated SLE	None identified	Normal/other
Zhang ZL, case 2	2009	PAPS	Other/unspecified trigger	Normal/other
Zhang ZL, case 3	2009	Secondary APS (SLE-associated)	Other/unspecified trigger	Hemorrhage-dominant
Zhang ZL, case 4	2009	Other CTD-associated APS	None identified	Enlargement/mass
Zhang ZL, case 5	2009	Isolated SLE	Other/unspecified trigger	Normal/other
Zhang ZL, case 6	2009	Other CTD-associated APS	None identified	Enlargement/mass
Yong	2010	Secondary APS (SLE-associated)	Thrombosis event	Hemorrhage-dominant
Boneschansker	2012	PAPS	Thrombosis event	Hemorrhage-dominant
Ramon, case 1	2013	Secondary APS (SLE-associated)	Thrombosis event	Enlargement/mass
Ramon, case 2	2013	PAPS	Multiple triggers	Hemorrhage-dominant
Ramon, case 3	2013	PAPS	None identified	Enlargement/mass
Ramon, case 4	2013	PAPS	Infection	Hemorrhage-dominant
Ramon, case 5	2013	PAPS	Surgery/procedure	Hemorrhage-dominant
Ramon, case 6	2013	PAPS	Anticoagulation-related	Hemorrhage-dominant
Ramon, case 7	2013	PAPS	Anticoagulation-related	Hemorrhage-dominant
Ramon, case 8	2013	PAPS	Surgery/procedure	Enlargement/mass
Ramon, case 9	2013	Possible SLE-spectrum APS	Surgery/procedure	Enlargement/mass
Ramon, case 10	2013	Secondary APS (SLE-associated)	Multiple triggers	Hemorrhage-dominant
Ramon, case 11	2013	PAPS	Pregnancy/postpartum	Necrosis/chronic change
Ramon, case 12	2013	PAPS	Other/unspecified trigger	Hemorrhage-dominant
Ramon, case 13	2013	PAPS	None identified	Hemorrhage-dominant
Ramon, case 14	2013	PAPS	None identified	Hemorrhage-dominant
Ramon, case 15	2013	PAPS	None identified	Hemorrhage-dominant
Ramon, case 16	2013	PAPS	None identified	Hemorrhage-dominant
Khare	2017	PAPS	Other/unspecified trigger	Hemorrhage-dominant
Aldaajani	2018	Secondary APS (SLE-associated)	Anticoagulation-related	Hemorrhage-dominant
Kolinioti, case 1	2018	PAPS	Other/unspecified trigger	Hemorrhage-dominant
Kolinioti, case 2	2018	PAPS	Other/unspecified trigger	Hemorrhage-dominant
Minami	2018	PAPS	Other/unspecified trigger	Hemorrhage-dominant
Xu	2018	Secondary APS (SLE-associated)	Other/unspecified trigger	Hemorrhage-dominant
Sanford	2019	PAPS	Anticoagulation-related	Hemorrhage-dominant
Wang-2019	2019	Isolated SLE	Other/unspecified trigger	Hemorrhage-dominant
Bansal	2020	PAPS	Thrombosis event	Hemorrhage-dominant
Mlekus, case 1	2020	PAPS	None identified	Necrosis/chronic change
Mlekus, case 2	2020	Other CTD-associated APS	Infection	Necrosis/chronic change
Warriach	2020	PAPS	Other/unspecified trigger	Enlargement/mass
Aneke	2021	PAPS	Anticoagulation-related	Hemorrhage-dominant
Jacobs	2021	PAPS	Anticoagulation-related	Hemorrhage-dominant
Billet	2022	PAPS	Infection	Hemorrhage-dominant
Ochi	2022	PAPS	Other/unspecified trigger	Hemorrhage-dominant
Tan	2022	PAPS	Anticoagulation-related	Hemorrhage-dominant
Bouki	2023	Secondary APS (SLE-associated)	Other/unspecified trigger	Hemorrhage-dominant
Diana	2023	Secondary APS (SLE-associated)	Thrombosis event	Hemorrhage-dominant
Jiang	2023	Secondary APS (SLE-associated)	Anticoagulation-related	Enlargement/mass
Ruizet	2023	PAPS	Other/unspecified trigger	Hemorrhage-dominant
Alameri	2024	PAPS	Other/unspecified trigger	Hemorrhage-dominant
Gao, case 1	2024	Secondary APS (SLE-associated)	Other/unspecified trigger	Normal/other
Gao, case 2	2024	Secondary APS (SLE-associated)	Other/unspecified trigger	Normal/other
Gao, case 3	2024	Secondary APS (SLE-associated)	Other/unspecified trigger	Normal/other
Gao, case 4	2024	Secondary APS (SLE-associated)	Other/unspecified trigger	Normal/other
Gao, case 5	2024	Isolated SLE	None identified	Normal/other
Gao, case 6	2024	Isolated SLE	None identified	Normal/other
Goel	2024	PAPS	Infection	Enlargement/mass
Liang	2024	Secondary APS (SLE-associated)	Other/unspecified trigger	Infarction-dominant
Mendiola	2024	Secondary APS (SLE-associated)	Infection	Hemorrhage-dominant
Pokharel	2024	PAPS	Infection	Normal/other
Shoji	2024	Secondary APS (SLE-associated)	Other/unspecified trigger	Infarction-dominant
Abdullah	2025	Isolated SLE	Multiple triggers	Hemorrhage-dominant
Abu Keshek	2025	Isolated SLE	None identified	Imaging unclear
Chung	2025	Secondary APS (SLE-associated)	Other/unspecified trigger	Hemorrhage-dominant
Fischetti	2025	PAPS	Multiple triggers	Mixed hemorrhage/infarction
Wang-2025	2025	Secondary APS (SLE-associated)	Other/unspecified trigger	Hemorrhage-dominant

This table summarizes individual published patients in whom adrenal insufficiency or Addison disease represented the sentinel presentation of APS or SLE-spectrum disease. To preserve readability, only patient reference, year, diagnosis category, precipitating trigger group, and adrenal imaging phenotype are shown. Detailed immunologic, electrolyte, CAPS, and outcome variables are summarized elsewhere in the pooled dataset.

Phenotypic transitions were evaluated across trigger context, imaging pattern, adrenal chronology, and severe presentation/follow-up category.

**Figure 3:**
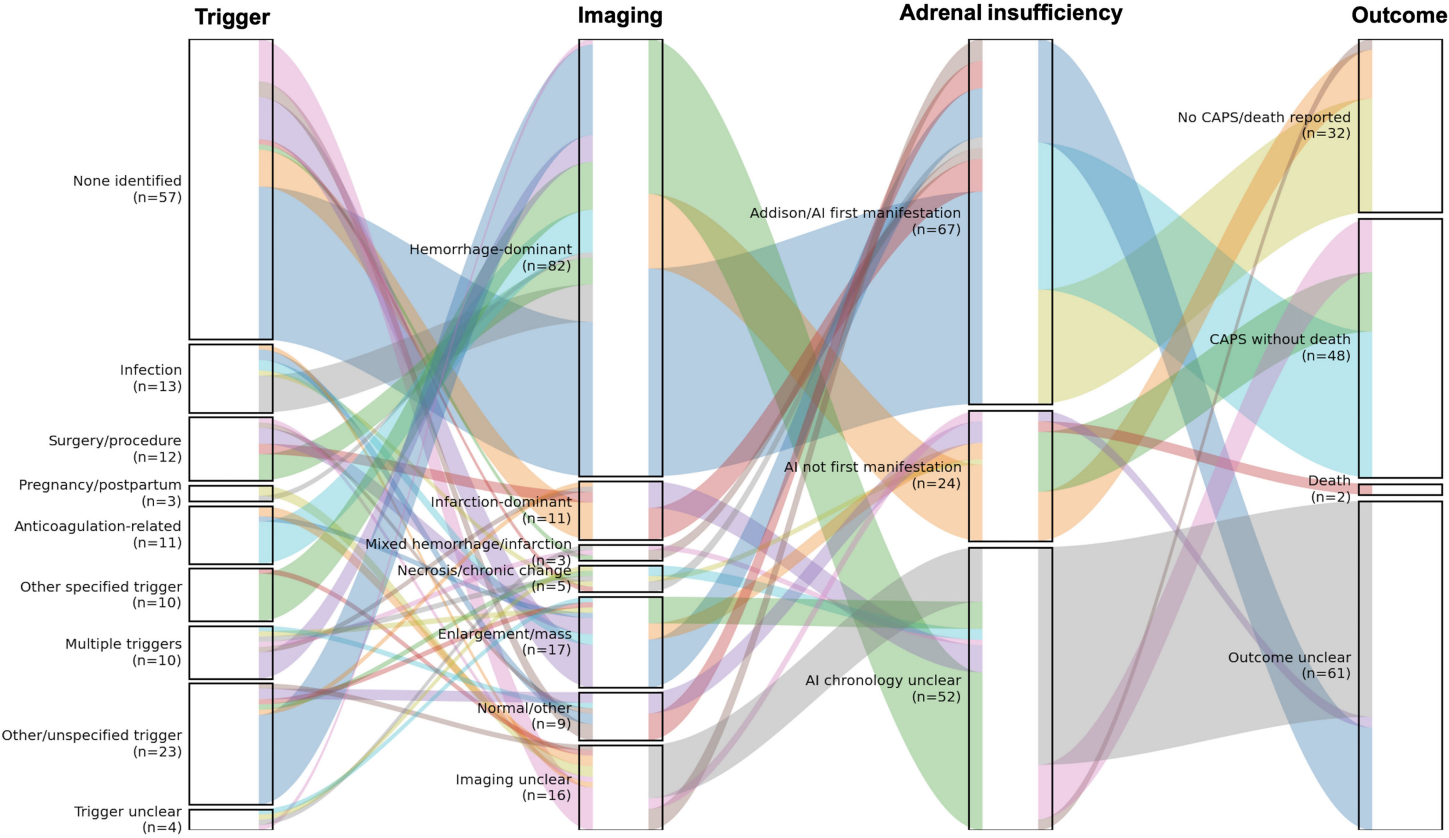
Phenotypic transitions across trigger, imaging pattern, adrenal chronology, and severe presentation/follow-up category. Alluvial plot showing the flow of cases from precipitating factor group to adrenal imaging phenotype, adrenal insufficiency chronology, and final severe presentation/follow-up category. For visualization, non-mutually exclusive clinical variables were collapsed into mutually exclusive display categories. The figure highlights dominant phenotypic pathways linking trigger context, adrenal vascular phenotype, and severe presentation and follow-up categories.

The alluvial phenotype map in **Figure 3** illustrates the flow of cases from the precipitating trigger group to the adrenal imaging phenotype, adrenal insufficiency chronology, and final severe presentation/follow-up category. The visually dominant pathway linked absent or nonspecific triggers to hemorrhage-dominant adrenal disease, followed by adrenal insufficiency occurring as an early or first manifestation. Overall, the figure shows that multiple upstream clinical contexts converged on a relatively limited set of high-risk adrenal vascular phenotypes.

**Figure 4:**
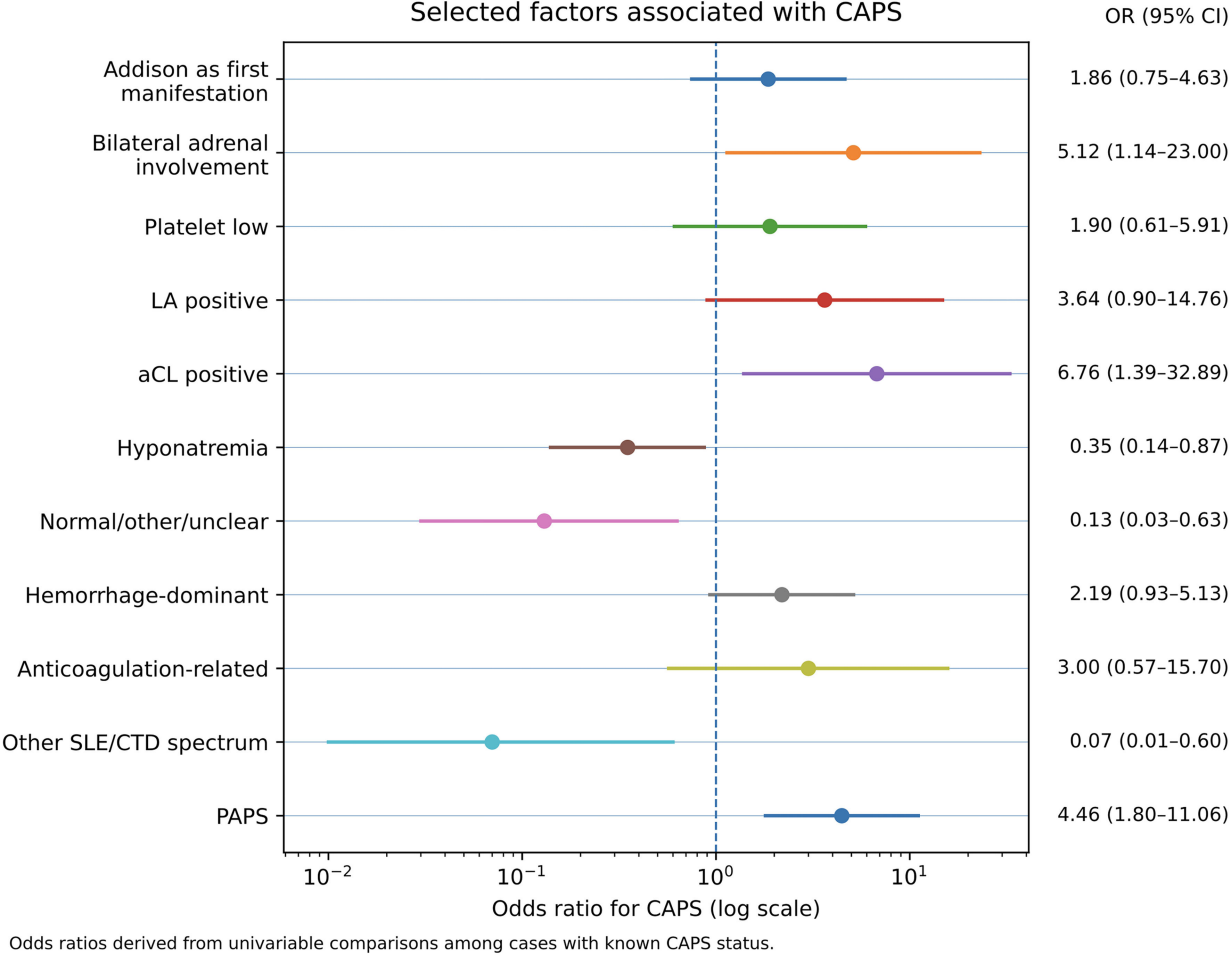
Exploratory univariable associations of selected case-level factors with CAPS presentation/context. Forest plot showing unadjusted odds ratios with 95% confidence intervals for selected diagnostic, trigger, imaging, and clinical/laboratory variables in CAPS context versus non-CAPS cases. Estimates were derived from available-case univariable comparisons among patients with known CAPS status. The vertical reference line indicates an odds ratio of 1. Because of sparse data and incomplete case-level reporting, these estimates should be interpreted as exploratory.

**Figure 5:**
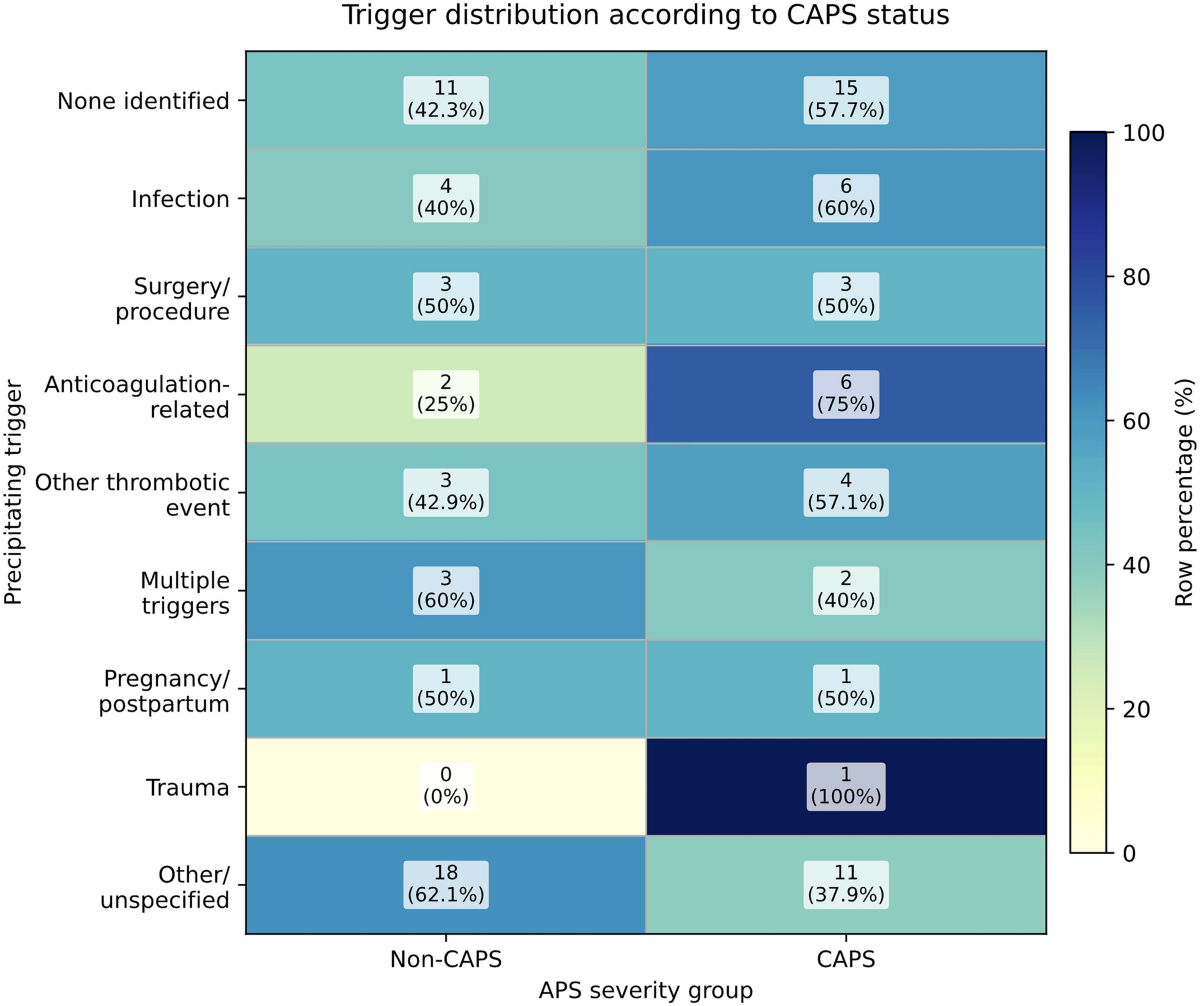
Trigger distribution according to CAPS presentation/context. Heatmap showing the distribution of precipitating trigger categories in CAPS context and non-CAPS cases with known CAPS status. Cell labels indicate case counts and row percentages. The figure emphasizes the relative trigger profile within each category rather than absolute prevalence in the full pooled cohort.

Exploratory univariable associations with CAPS presentation/context are summarized in **Table 4** and visualized in **Figures 4**, **5**. These analyses were restricted to the 94 patients with known CAPS status. They should be interpreted as descriptive signals of a severe presentation context rather than evidence that CAPS is a consequence of adrenal involvement.

**Table 4:** Exploratory univariable associations of selected case-level factors with CAPS presentation/context.

Section	Variable	Total	CAPS	Non-CAPS	OR (95% CI)	*P*-value
Diagnosis	PAPS	60/94 (63.8%)	39/49 (79.6%)	21/45 (46.7%)	4.46 (1.80–11.06)	0.001
	Secondary APS (SLE-associated)	23/94 (24.5%)	9/49 (18.4%)	14/45 (31.1%)	0.50 (0.19–1.30)	0.230
	Other SLE/CTD spectrum	11/94 (11.7%)	1/49 (2.0%)	10/45 (22.2%)	0.07 (0.01–0.60)	0.003
Trigger	None identified	26/94 (27.7%)	15/49 (30.6%)	11/45 (24.4%)	1.36 (0.55–3.39)	0.645
	Infection	10/94 (10.6%)	6/49 (12.2%)	4/45 (8.9%)	1.43 (0.38–5.44)	0.742
	Surgery/procedure	6/94 (6.4%)	3/49 (6.1%)	3/45 (6.7%)	0.91 (0.17–4.77)	1.000
	Anticoagulation-related	8/94 (8.5%)	6/49 (12.2%)	2/45 (4.4%)	3.00 (0.57–15.70)	0.271
	Thrombosis event	7/94 (7.4%)	4/49 (8.2%)	3/45 (6.7%)	1.24 (0.26–5.89)	1.000
	Multiple triggers	5/94 (5.3%)	2/49 (4.1%)	3/45 (6.7%)	0.60 (0.09–3.74)	0.668
	Other specific/unspecified trigger	32/94 (34.0%)	13/49 (26.5%)	19/45 (42.2%)	0.49 (0.21–1.18)	0.130
Imaging	Hemorrhage-dominant	59/94 (62.8%)	35/49 (71.4%)	24/45 (53.3%)	2.19 (0.93–5.13)	0.089
	Infarction-dominant	6/94 (6.4%)	4/49 (8.2%)	2/45 (4.4%)	1.91 (0.33–10.98)	0.679
	Enlargement/mass	11/94 (11.7%)	4/49 (8.2%)	7/45 (15.6%)	0.48 (0.13–1.77)	0.342
	Mixed or chronic change	5/94 (5.3%)	4/49 (8.2%)	1/45 (2.2%)	3.91 (0.42–36.39)	0.364
	Normal/other/unclear	13/94 (13.8%)	2/49 (4.1%)	11/45 (24.4%)	0.13 (0.03–0.63)	0.006
Clinical/ laboratory	Hyponatremia	29/87 (33.3%)	11/48 (22.9%)	18/39 (46.2%)	0.35 (0.14–0.87)	0.039
	Hyperkalemia	40/79 (50.6%)	25/47 (53.2%)	15/32 (46.9%)	1.29 (0.52–3.17)	0.650
	aCL-positive	80/92 (87.0%)	46/48 (95.8%)	34/44 (77.3%)	6.76 (1.39–32.89)	0.012
	LA-positive	78/89 (87.6%)	45/48 (93.8%)	33/41 (80.5%)	3.64 (0.90–14.76)	0.103
	β2GPI-positive	6/6 (100.0%)	3/3 (100.0%)	3/3 (100.0%)	1.00 (0.02–66.06)	1.000
	Low platelet	43/60 (71.7%)	27/35 (77.1%)	16/25 (64.0%)	1.90 (0.61–5.91)	0.384
	Bilateral adrenal involvement	57/66 (86.4%)	41/44 (93.2%)	16/22 (72.7%)	5.12 (1.14–23.00)	0.051
	Addison as first manifestation	50/81 (61.7%)	27/39 (69.2%)	23/42 (54.8%)	1.86 (0.75–4.63)	0.253
Outcomes	Death	2/11 (18.2%)	1/3 (33.3%)	1/8 (12.5%)	3.50 (0.14–84.69)	0.491
	Long-term replacement	4/5 (80.0%)	1/1 (100.0%)	3/4 (75.0%)	1.29 (0.03–53.51)	1.000
	Any adrenal recovery	4/5 (80.0%)	NA	4/5 (80.0%)	0.33 (0.00–25.41)	1.000

Data are shown as *n*/*N* (%) unless otherwise indicated. Odds ratios (ORs) with 95% confidence intervals (CIs) were derived from univariable case-level comparisons between CAPS and non-CAPS groups among cases with known CAPS status. Denominators vary across variables because of incomplete reporting in published case reports. These associations should be interpreted as exploratory because of sparse cells and substantial missingness.

CAPS, catastrophic antiphospholipid syndrome; aCL, anticardiolipin antibodies; LA, lupus anticoagulant; β2GPI, anti-β2 glycoprotein I; CTD, connective tissue disease.

### Multivariable analysis

The multivariable Firth logistic regression models were moved to the **Supplementary Appendix** because they are exploratory and complete-case-based. The model estimates are provided in **Supplementary Table 2** and displayed in **Supplementary Figure 1**. In brief, the direction of association for LA positivity and bilateral adrenal involvement was positive but imprecise, with wide confidence intervals. These models are retained only as bias-reduced signal analyses and not as causal or prognostic models.

### Proposed clinical recognition framework

**Figure 6:**
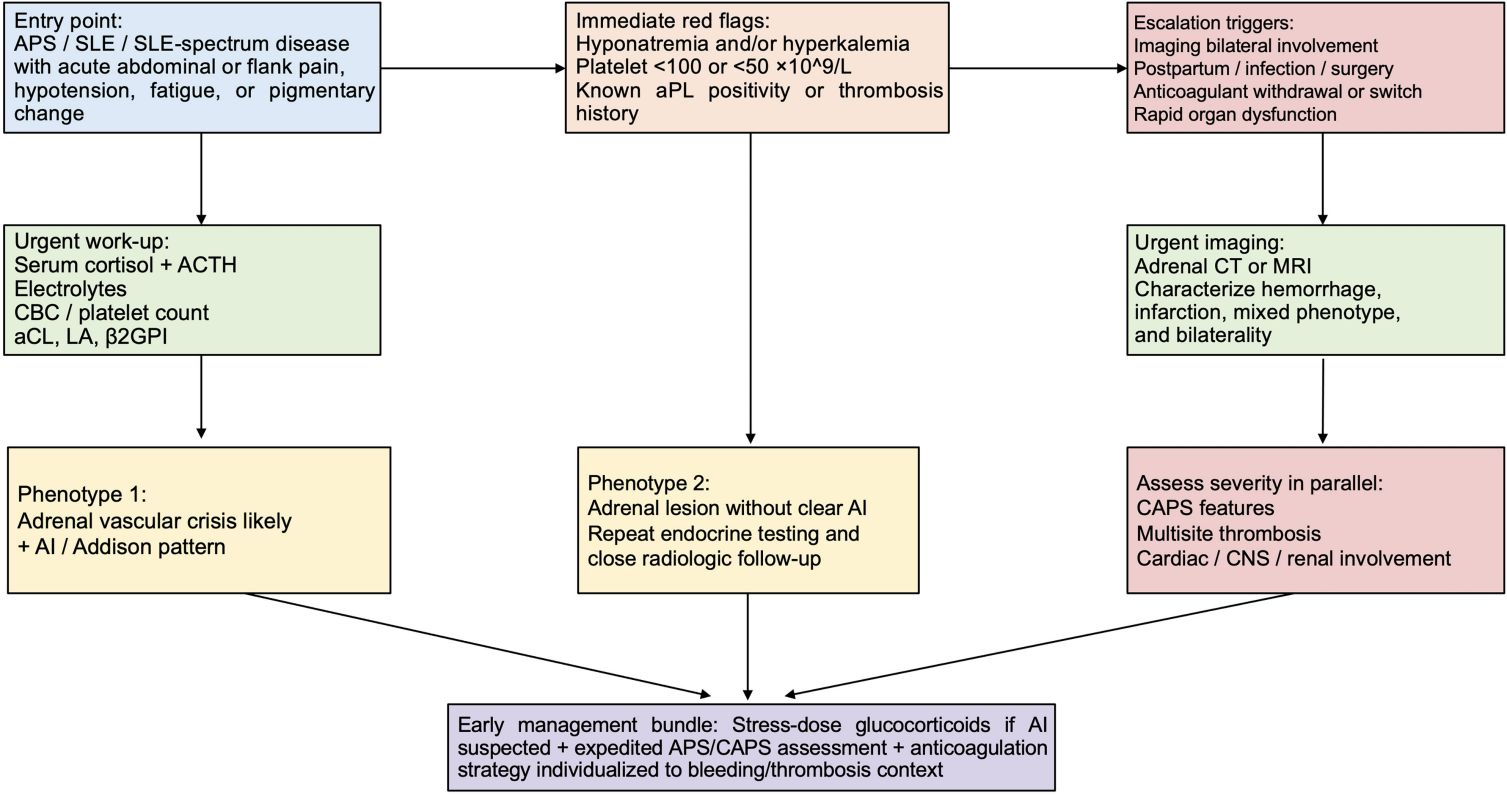
Proposed clinical recognition framework for adrenal involvement in APS and SLE-spectrum disease. Proposed clinical recognition framework for adrenal involvement in APS, SLE, and SLE-spectrum disease. The framework integrates clinical entry points, red-flag features, endocrine evaluation, adrenal imaging, severity assessment, and early management priorities. The algorithm is intended as a pragmatic recognition tool derived from pooled published cases rather than a validated decision rule.

Based on the pooled case data, we developed a pragmatic clinical recognition framework for adrenal involvement in APS, SLE, and SLE-spectrum disease (**Figure 6**). The framework integrates typical clinical entry points, red-flag features, endocrine testing, adrenal imaging, assessment for systemic thrombosis/CAPS presentation context, and early management priorities. It is intended to translate the pooled case-level phenotype into a clinically usable recognition pathway rather than to serve as a validated decision rule.

### Overall interpretation of the pooled case series

Taken together, these findings indicate that adrenal involvement in APS and SLE-spectrum disease is not a single uniform entity but rather a heterogeneous, high-risk clinicoradiologic syndrome shaped by trigger context, adrenal imaging phenotype, immunologic background, and systemic thrombotic burden. The pooled case literature was dominated by APS-defined disease, especially primary APS, and most informative cases involved bilateral adrenal disease with hemorrhagic predominance. At the same time, sentinel-presentation cases and CAPS-context summaries show that adrenal insufficiency may be both an entry point into diagnosis and a marker of systemic thrombotic severity.

## Discussion

In this systematic review and pooled individual-patient analysis, adrenal involvement emerged as a predominantly APS-defined, bilateral, and hemorrhage-dominant phenotype, with a substantial burden of CAPS presentation/context and a strikingly high frequency of adrenal insufficiency as the first clinically recognized manifestation of disease. These findings complement the 86-patient APS adrenal series by Espinosa et al. and the 256-patient mixed multicenter/systematic review by Meade-Aguilar et al. while broadening the scope to SLE-spectrum diagnostic strata and case-level trigger, chronology, and follow-up harmonization (6, 11).

Our imaging results are biologically plausible. The predominance of bilateral involvement and hemorrhage-dominant lesions is consistent with the long-standing concept that the adrenal gland is especially susceptible to venous outflow obstruction, microthrombosis, and secondary hemorrhagic infarction (9). The 2023 ACR/EULAR APS criteria now recognize adrenal hemorrhage or microthrombosis within the microvascular clinical domain, reinforcing that adrenal involvement should be conceptualized as a vascular APS manifestation rather than as isolated endocrine disease (10).

The trigger distribution also deserves attention. Although many published cases lacked a clearly documented precipitating factor, infection, surgery/procedure, anticoagulation-related contexts, thrombosis events, and pregnancy/postpartum states recurred across the cohort and were visually linked to severe downstream trajectories in the phenotype map. This aligns with CAPS literature, in which infections and procedural stress are established precipitants of multisite thrombotic collapse (2, 8, 113). The relative enrichment of anticoagulation-related cases among CAPS patients in our exploratory analyses is particularly clinically relevant because hemorrhagic adrenal lesions can misleadingly suggest that anticoagulation is causative rather than that an underlying thrombotic adrenal event has progressed to secondary hemorrhage.

One of the clearest practical signals in our dataset was the frequency with which adrenal insufficiency or Addison disease represented the sentinel clinical manifestation. This observation reinforces prior reports that primary adrenal failure can be the heralding event of APS and occasionally of secondary APS in SLE (5–8, 15). In practice, bilateral adrenal enlargement, hemorrhage, infarction, or otherwise unexplained adrenal abnormalities accompanied by hypotension, abdominal or flank pain, hyponatremia, hyperkalemia, thrombocytopenia, or a positive aPL profile should prompt immediate consideration of APS/SLE-spectrum disease. Importantly, the absence of the classic electrolyte pattern should not be reassuring because endocrine features were incompletely reported and not all informative cases showed fully evolved biochemical adrenal failure at presentation.

Although bilateral adrenal involvement was the dominant pattern, unilateral adrenal lesions should not be dismissed. A small number of source reports described adrenal insufficiency in the setting of unilateral imaging findings or sequential adrenal involvement. Possible explanations include early contralateral involvement below imaging sensitivity, prior subclinical contralateral damage, hemorrhagic/infarctive evolution between scans, severe systemic illness lowering adrenal reserve, or incomplete imaging documentation. We therefore regard unilateral adrenal abnormalities in APS/SLE-spectrum disease as clinically relevant when accompanied by hypotension, electrolyte disturbance, low cortisol/elevated ACTH, or aPL positivity.

The immunologic profile of the pooled cohort was dominated by aCL and LA positivity, while ANA positivity and anti-dsDNA positivity identified the expected SLE-enriched subset. This pattern supports the concept that adrenal involvement lies at the intersection of autoantibody positivity, endothelial injury, and systemic thrombosis rather than within a single disease label alone (1–3, 113). A complementary mechanistic layer is provided by the lipid-raft signaling model of APS. Capozzi et al. summarized evidence that aPLs are not merely serologic markers but can activate cells, coagulation factors, and inflammatory mediators; these effects require interactions between aPLs and target receptors within cell-membrane lipid-raft microdomains. Anti-β2-glycoprotein I antibodies may engage β2-glycoprotein I, annexin A2, TLR2/TLR4, and related signaling complexes within lipid rafts of endothelial cells, monocytes, and platelets, thereby promoting NF-κB-dependent inflammation, tissue factor expression, and a procoagulant phenotype (114). Although direct lipid-raft-mediated injury in adrenal cortical cells has not been specifically demonstrated in the published adrenal cases, this pathway provides a biologically plausible complement to the venous thrombosis and hemorrhagic infarction model. It may help explain how systemic aPL-mediated endothelial and microvascular activation could predispose to adrenal microthrombosis, hemorrhagic infarction, and adrenal dysfunction, including cases in which macroscopic imaging is unilateral, delayed, or incomplete.

Two findings from the univariable CAPS analysis warrant cautious interpretation. First, hyponatremia appeared less frequent among CAPS cases. Given the heavy missingness and uneven reporting across case reports, this is more plausibly a reporting artifact or timing effect than a protective biological signal. Second, normal/other/unclear imaging categories were inversely associated with CAPS, likely because severe cases were more likely to undergo more definitive imaging or to display overt destructive adrenal lesions. These observations illustrate why available-case case-report meta-data can identify plausible signals but cannot substitute for uniformly measured prospective cohorts.

Our dataset also captured a contemporary treatment-context phenotype that deserves emphasis. Although the absolute numbers were small, recent SLE/APS reports suggest that thrombopoietin receptor agonist exposure in aPL-positive immune thrombocytopenia may coexist with serious thrombotic complications, including adrenal hemorrhage, thrombotic microangiopathy, and CAPS (7, 13–16). The fatal case reported by Chung and Ha illustrates the extreme end of this spectrum, while earlier reports by Guitton et al., LaMoreaux et al., and Tomov et al. highlight the same concern in different clinical settings (12–14, 16). These data are not sufficient to define causality, but they do support heightened thrombotic vigilance when thrombopoietin-receptor agonists are used in patients with SLE and persistent aPL.

From a management perspective, the pooled data argue for an integrated endocrine–rheumatologic–thrombosis approach. Immediate glucocorticoid replacement is essential when adrenal insufficiency is suspected, but evaluation should not stop at endocrine rescue. Mineralocorticoid replacement should be considered when primary adrenal insufficiency is confirmed or strongly suspected, although published case reports often did not separate glucocorticoid from mineralocorticoid treatment. aPL testing, assessment for bilateral adrenal involvement, and active screening for extra-adrenal thrombosis or CAPS presentation context are crucial because the adrenal lesion may be only one component of a broader systemic event (94, 113).

This study has several strengths. It provides a comprehensive individual-patient synthesis across APS and SLE-spectrum disease, with explicit harmonization of trigger context, adrenal chronology, imaging phenotype, symptoms, hemodynamic status, and follow-up variables. The study also has important limitations. All included data were retrospective and case-based, making the pooled cohort vulnerable to publication bias, selection toward dramatic presentations, inconsistent variable definition, and substantial missingness. Denominators varied across analyses, non-APS strata were small, treatment/follow-up imaging details were often unavailable, and the multivariable models were complete-case exploratory analyses reported only in **Supplementary Table 2** and **Supplementary Figure 1**. We did not independently re-read the original adrenal imaging, and extraction relied on source-report descriptions.

In conclusion, adrenal involvement in APS and SLE-spectrum disease is a heterogeneous but clinically recognizable immunothrombotic syndrome characterized by a strong predilection for bilateral hemorrhagic adrenal injury, frequent sentinel presentation as adrenal insufficiency, and substantial CAPS presentation burden. Early recognition is likely to improve both endocrine rescue and identification of the underlying systemic autoimmune-thrombotic disorder.

## Data Availability

The original contributions presented in the study are included in the article/**Supplementary Material**, further inquiries can be directed to the corresponding author/s.
