# Supplementary material for: Adrenal involvement in antiphospholipid syndrome and SLE-spectrum disease: a systematic review and pooled individual-patient analysis
Source: Front Immunol. 2026 Jun 3;17:1851029. doi: 10.3389/fimmu.2026.1851029 (PMC13272318; doi:10.3389/fimmu.2026.1851029)
Supplement: Supplementary file 1 [file DataSheet1.pdf]

Supplementary Appendix 1. Search Strategy, Reviewer Process, Definitions, Diagnosis-Stratum Comparison, and Exploratory Multivariable Models

1. Database search strategy

Initial searches were run from database inception to 10 February 2026 in PubMed, Embase, and Web of Science. The searches were updated through 31 March 2026. The update did not add any new eligible full-text primary case report or case series to the final extraction set.

PubMed: ((("Antiphospholipid Syndrome"[Mesh]) OR "antiphospholipid syndrome*"[tiab] OR "Hughes syndrome*"[tiab] OR anticardiolipin[tiab] OR "lupus anticoagulant"[tiab] OR "anti-beta 2 glycoprotein I"[tiab] OR "anti-β2 glycoprotein I"[tiab]) AND (adrenal[tiab] OR suprarenal[tiab] OR "Adrenal Glands"[Mesh]) AND (hemorrhag*[tiab] OR haemorrhag*[tiab] OR infarct*[tiab] OR thrombos*[tiab] OR microthrombos*[tiab] OR necros*[tiab] OR insufficien*[tiab] OR failure[tiab] OR Addison*[tiab])) OR (((systemic lupus erythematosus[tiab] OR SLE[tiab] OR lupus[tiab] OR "Lupus Erythematosus, Systemic"[Mesh]) AND (adrenal[tiab] OR suprarenal[tiab]) AND (hemorrhag*[tiab] OR haemorrhag*[tiab] OR infarct*[tiab] OR thrombos*[tiab] OR insufficien*[tiab] OR Addison*[tiab])))

Embase: ('antiphospholipid syndrome'/exp OR 'antiphospholipid syndrome*':ti,ab OR 'hughes syndrome*':ti,ab OR anticardiolipin:ti,ab OR 'lupus anticoagulant':ti,ab OR 'anti beta 2 glycoprotein i':ti,ab) AND ('adrenal gland'/exp OR adrenal:ti,ab OR suprarenal:ti,ab) AND ('adrenal hemorrhage'/exp OR 'adrenal insufficiency'/exp OR hemorrhag*:ti,ab OR haemorrhag*:ti,ab OR infarct*:ti,ab OR thrombos*:ti,ab OR microthrombos*:ti,ab OR necros*:ti,ab OR insufficien*:ti,ab OR addison*:ti,ab) OR (('systemic lupus erythematosus'/exp OR 'systemic lupus erythematosus':ti,ab OR lupus:ti,ab OR SLE:ti,ab) AND ('adrenal gland'/exp OR adrenal:ti,ab OR suprarenal:ti,ab) AND (hemorrhag*:ti,ab OR haemorrhag*:ti,ab OR infarct*:ti,ab OR thrombos*:ti,ab OR insufficien*:ti,ab OR addison*:ti,ab))

Web of Science: TS=(("antiphospholipid syndrome" OR "Hughes syndrome" OR anticardiolipin OR "lupus anticoagulant" OR "anti-beta 2 glycoprotein I" OR "anti-β2 glycoprotein I") AND (adrenal OR suprarenal) AND (hemorrhag* OR haemorrhag* OR infarct* OR thrombos* OR microthrombos* OR necros* OR insufficien* OR failure OR Addison*)) OR TS=((lupus OR "systemic lupus erythematosus" OR SLE) AND (adrenal OR suprarenal) AND (hemorrhag* OR haemorrhag* OR infarct* OR thrombos* OR insufficien* OR Addison*))

Note: Addison disease/Addison* was retained only as a historical label used by older case reports for primary adrenal insufficiency. Cases of classic autoimmune Addison disease without APS/SLE-spectrum disease and vascular adrenal involvement were excluded.

Update-search note: the March 31, 2026 search was performed to reconcile the manuscript search cutoff and did not identify additional eligible full-text primary case reports or case series. Any noneligible records found during the update were screened using the same eligibility framework and did not change the final case-level extraction set.

2. Dual-review process and inter-rater agreement

Title/abstract screening, full-text eligibility assessment, and key case-level extraction were performed independently by Sheng-Guang Li and Ting Long. Disagreements were resolved by discussion and senior review of the source report.

Stage	Reviewer A selected/included	Reviewer A excluded	Reviewer B selected/included	Reviewer B excluded	Discordant decisions before consensus	Observed agreement	Cohen's kappa
Title/abstract screening (n=1,024)	109	915	108	916	15	98.5%	0.923
Full-text eligibility assessment (n=107)	103	4	102	5	1	99.1%	0.884
CAPS classification audit among extracted cases (n=155)	25 yes / 130 no	NA	24 yes / 131 no	NA	3	98.1%	0.927

For continuous variables such as cortisol, ACTH, age, and platelet count, Cohen's kappa was not calculated because the variables were numeric or source-text based; values were independently checked against the source report and reconciled by consensus.

3. Operational definitions and diagnostic strata

Variable / stratum	Operational definition used in revision	Caveat / source hierarchy
Definite APS	Source-level clinical APS features with reported aPL positivity compatible with revised Sapporo APS classification; interpreted in light of 2023 ACR/EULAR APS concepts when appropriate.	Many older reports predated modern classification and repeat-testing conventions.
Probable APS	Strong clinical APS phenotype but incomplete laboratory persistence documentation or incomplete source-level reporting.	Used for case-level harmonization, not as formal clinical diagnosis.
Non-APS SLE-spectrum disease	SLE/CTD cases with adrenal vascular involvement but no source-level APS classification.	Kept separate from definite/probable APS in primary analyses.
Primary APS	APS without source-reported SLE or another connective tissue disease.	Based on source report.
Secondary APS (SLE-associated)	APS occurring in a patient with source-reported SLE.	Based on source report and reported immunologic/clinical features.
Isolated SLE	SLE with adrenal involvement but without source-level APS diagnosis.	Not treated as APS unless aPL/clinical criteria supported APS.
Other CTD-associated APS	APS associated with a CTD other than SLE.	Sparse subgroup; descriptive only.
Possible SLE-spectrum APS	Cases with lupus-spectrum features and aPL/APS-like adrenal vascular phenotype but insufficient detail for definite classification.	Sensitivity/descriptive category only.
Hyponatremia	Source-reported hyponatremia or sodium <135 mmol/L when numeric values were available.	Numeric values not always reported.
Hyperkalemia	Source-reported hyperkalemia or potassium >5.0 mmol/L when numeric values were available.	Numeric values not always reported.
Adrenal insufficiency	Source-reported primary adrenal insufficiency/Addison disease, low cortisol with elevated ACTH, or clinical adrenal steroid replacement.	Classic autoimmune Addison disease without APS/SLE-spectrum disease and vascular adrenal involvement was excluded.
Strict adrenal crisis	Adrenal insufficiency accompanied by hypotension, shock, circulatory collapse, or explicit source diagnosis of adrenal crisis.	Broader adrenal insufficiency alone was not coded as adrenal crisis.
CAPS presentation/context	Source-reported CAPS or published criteria-compatible catastrophic APS when sufficient source detail was available.	Analyzed as presentation/severity context, not downstream adrenal outcome.
aPL positivity	Source-reported positivity for LA, aCL, anti-β2GPI, or other aPL assays.	Criterion-level/persistent positivity retained when explicitly documented.
Triple positivity	Concurrent positivity for LA, aCL, and anti-β2GPI when all three domains were reported.	Underreported in older literature.
Hypotension	Source text contained hypotension, low blood pressure, low BP, orthostatic/postural hypotension, or comparable hemodynamic wording.	No numeric BP parsing was possible unless present in source text.
Glucocorticoid replacement	Hydrocortisone, cortisone acetate, prednisone/prednisolone, methylprednisolone, dexamethasone, glucocorticoid, steroid replacement, or equivalent adrenal replacement wording when explicitly treatment-related.	Nonspecific steroid use for SLE/CAPS was not automatically counted as adrenal replacement.
Mineralocorticoid replacement	Fludrocortisone or explicit mineralocorticoid replacement.	Often omitted in source reports.
Follow-up adrenal imaging	Follow-up CT/MRI/ultrasound or source-reported radiologic change in adrenal lesion size, hematoma resolution, atrophy, or persistence.	Endocrine recovery without imaging was not coded as follow-up imaging.
Source hierarchy	Primary case report full text > open-access article/PMC > PubMed abstract > table in recent systematic review > title/snippet only.	Review-table extraction could guide verification but did not override primary reports when available.

4. Supplementary tables and figures

Supplementary Table S1. Comparison of clinical, immunologic, imaging, and outcome characteristics by diagnosis stratum.

Characteristic	PAPS	Secondary APS (SLE-associated)	Isolated SLE	Other CTD-associated APS	Possible SLE-spectrum APS	Explora-tory p overall
Cases, n	96	36	5	4	2	
Female sex	41 (42.7%)	23 (63.9%)	3 (60.0%)	2 (50.0%)	1 (50.0%)	0.292
Age, years	45.0 (33.0–54.0)	43.0 (29.5–55.0)	35.0 (32.0–49.0)	45.0 (34.0–57.2)	30.4 (30.2–30.7)	0.536
Age ≥50 years	34 (35.4%)	13 (36.1%)	1 (20.0%)	2 (50.0%)	0 (0.0%)	0.737
Trigger information available	94 (100.0%)	34 (100.0%)	5 (100.0%)	4 (100.0%)	2 (100.0%)	NR
Infection	11 (11.7%)	3 (8.8%)	0 (0.0%)	1 (25.0%)	0 (0.0%)	0.754
Surgery/ procedure	11 (11.7%)	4 (11.8%)	1 (20.0%)	0 (0.0%)	2 (100.0%)	0.006
Pregnancy/ postpartum	4 (4.3%)	1 (2.9%)	1 (20.0%)	0 (0.0%)	1 (50.0%)	0.022
Anticoagulation withdrawal	8 (8.5%)	3 (8.8%)	0 (0.0%)	0 (0.0%)	0 (0.0%)	0.905
Hyponatremia	30 (31.2%)	14 (40.0%)	2 (40.0%)	1 (25.0%)	0 (0.0%)	0.714
Hyperkalemia	38 (40.4%)	17 (51.5%)	1 (33.3%)	1 (50.0%)	0 (0.0%)	0.576
CAPS	39 (68.4%)	9 (52.9%)	0 (0.0%)	1 (33.3%)	0 (0.0%)	0.034
Addison/AI as first manifestation	48 (80.0%)	17 (77.3%)	0 (0.0%)	0 (0.0%)	2 (100.0%)	<0.001
aCL positive	85 (94.4%)	32 (97.0%)	0 (0.0%)	2 (50.0%)	2 (100.0%)	<0.001
LA positive	82 (91.1%)	33 (94.3%)	2 (40.0%)	1 (33.3%)	2 (100.0%)	<0.001
ANA positive	31 (37.8%)	29 (85.3%)	4 (100.0%)	1 (33.3%)	0 (0.0%)	<0.001
Anti-dsDNA positive	0 (0.0%)	25 (78.1%)	2 (66.7%)	0 (0.0%)	0 (0.0%)	<0.001
β2GPI positive	1 (100.0%)	5 (100.0%)	NR	NR	NR	NR
Platelet <100 ×10^9/L	NR	NR	1 (100.0%)	NR	NR	NR
Bilateral adrenal involvement	54 (85.7%)	24 (88.9%)	2 (66.7%)	2 (100.0%)	NR	NR
Hemorrhage phenotype	59 (70.2%)	23 (71.9%)	3 (60.0%)	0 (0.0%)	0 (0.0%)	0.011
Infarction phenotype	11 (13.1%)	3 (9.4%)	0 (0.0%)	0 (0.0%)	0 (0.0%)	0.769
Any extra-adrenal thrombosis	4 (100.0%)	3 (100.0%)	1 (100.0%)	NR	NR	NR
Multisite thrombosis	2 (50.0%)	2 (66.7%)	1 (100.0%)	NR	NR	NR
Long-term adrenal replacement	1 (100.0%)	2 (100.0%)	NR	NR	NR	NR
Any adrenal recovery	1 (100.0%)	2 (66.7%)	NR	NR	NR	NR
Death	0 (0.0%)	1 (16.7%)	1 (100.0%)	NR	NR	NR
Severe outcome (death or CAPS)	39 (68.4%)	9 (52.9%)	1 (25.0%)	1 (33.3%)	0 (0.0%)	0.165

Data are presented as median (IQR) for continuous variables and n (%) for categorical variables. Percentages were calculated from available cases for each variable. Subgroup comparisons are descriptive because of sparse cells and uneven group sizes.

Supplementary Table S2. Multivariable Firth logistic regression models for CAPS presentation/context and severe composite outcome.

Outcome	Predictor	Adjusted OR	Lower 95% CI	Upper 95% CI	Adjusted OR (95% CI)	Complete-case N	Events
CAPS	Age ≥50 years	1.254815516	0.297104007	5.299699578	1.25(0.30–5.30)	51	41
CAPS	LA positive	4.011454783	0.656051983	24.52819271	4.01(0.66–24.53)	51	41
CAPS	Bilateral adrenal involvement	2.72443298	0.36268356	20.4655956	2.72(0.36–20.47)	51	41
CAPS	Infection trigger	1.663017628	0.057429992	48.15650374	1.66(0.06–48.16)	51	41
Severe composite outcome (death or CAPS)	Age ≥50 years	1.00952139	0.225866494	4.512105435	1.01(0.23–4.51)	51	42
Severe composite outcome (death or CAPS)	LA positive	4.852049099	0.76462867	30.78929861	4.85(0.76–30.79)	51	42
Severe composite outcome (death or CAPS)	Bilateral adrenal involvement	3.062867271	0.392756686	23.88541365	3.06(0.39–23.89)	51	42
Severe composite outcome (death or CAPS)	Infection trigger	1.470225918	0.050181368	43.07503623	1.47(0.05–43.08)	51	42

Adjusted odds ratios with 95% confidence intervals were estimated using Firth logistic regression with complete-case analysis. These models are retained only as bias-reduced exploratory signal analyses and not as causal or prognostic models.

Supplementary Figure S1. Multivariable bias-reduced logistic regression models for CAPS presentation/context and severe composite outcome.

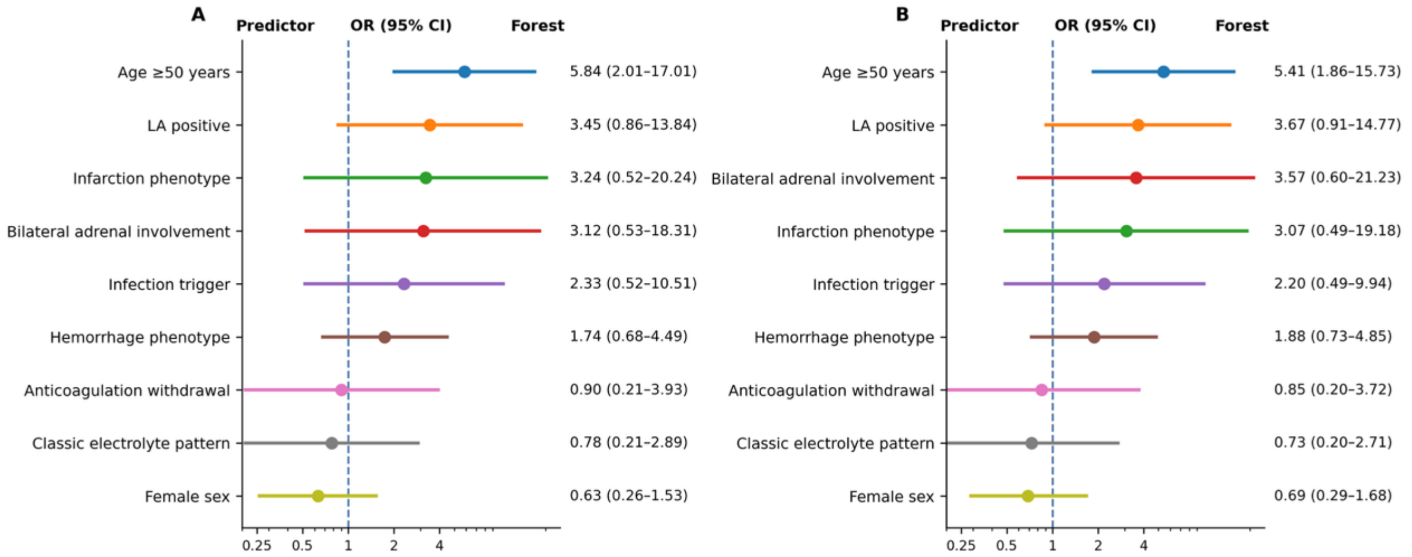

Forest plots showing adjusted odds ratios with 95% confidence intervals derived from multivariable Firth logistic regression. Panel A shows predictors of CAPS. Panel B shows predictors of severe composite outcome, defined as death or CAPS.
